# Association of Odor Thresholds and Responses in Cerebral Blood Flow of the Prefrontal Area during Olfactory Stimulation in Patients with Multiple Chemical Sensitivity

**DOI:** 10.1371/journal.pone.0168006

**Published:** 2016-12-09

**Authors:** Kenichi Azuma, Iwao Uchiyama, Mari Tanigawa, Ikuko Bamba, Michiyo Azuma, Hirohisa Takano, Toshikazu Yoshikawa, Kou Sakabe

**Affiliations:** 1 Department of Environmental Medicine and Behavioral Science, Kindai University Faculty of Medicine, Osakasayama, Osaka, Japan; 2 Sick-house Medical Science Laboratory, Division of Basic Research, Louis Pasteur Center for Medical Research, Kyoto, Japan; 3 Outpatient Department of Sick-house Syndrome, Hyakumanben Clinic, Kyoto, Japan; 4 Clinical Immune Function Laboratory, Division of Basic Research, Louis Pasteur Center for Medical Research, Kyoto, Japan; 5 Division of Internal Medicine, Hyakumanben Clinic, Kyoto, Japan; 6 Faculty of Education, Home Economics, Tokyo Gakugei University, Koganei, Tokyo, Japan; 7 Department of Human Environmental Design, Faculty of Health Science, Kio University, Kitakatsuragi-gun, Nara, Japan; 8 Department of Environmental Engineering, Graduate School of Engineering, Kyoto University, Kyoto, Japan; 9 Department of Internal Medicine, Kyoto Prefectural University of Medicine, Kyoto, Japan; 10 Department of Anatomy and Cellular Biology, Tokai University School of Medicine, Isehara, Kanagawa, Japan; Duke University, UNITED STATES

## Abstract

Multiple chemical sensitivity (MCS) is a disorder characterized by nonspecific and recurrent symptoms from various organ systems associated with exposure to low levels of chemicals. Patients with MCS process odors differently than controls do. Previously, we suggested that this odor processing was associated with increased regional cerebral blood flow (rCBF) in the prefrontal area during olfactory stimulation using near-infrared spectroscopic (NIRS) imaging. The aim of this study was to investigate the association of odor thresholds and changes in rCBF during olfactory stimulation at odor threshold levels in patients with MCS. We investigated changes in the prefrontal area using NIRS imaging and a T&T olfactometer during olfactory stimulation with two different odorants (sweet and fecal) at three concentrations (zero, odor recognition threshold, and normal perceived odor level) in 10 patients with MCS and six controls. The T&T olfactometer threshold test and subjective assessment of irritating and hedonic odors were also performed. The results indicated that the scores for both unpleasant and pungent odors were significantly higher for those for sweet odors at the normal perceived level in patients with MCS than in controls. The brain responses at the recognition threshold (fecal odor) and normal perceived levels (sweet and fecal odors) were stronger in patients with MCS than in controls. However, significant differences in the odor detection and recognition thresholds and odor intensity score between the two groups were not observed. These brain responses may involve cognitive and memory processing systems during past exposure to chemicals. Further research regarding the cognitive features of sensory perception and memory due to past exposure to chemicals and their associations with MCS symptoms is needed.

## Introduction

Multiple chemical sensitivity (MCS) is a disorder characterized by nonspecific and recurrent symptoms from various organ systems associated with exposure to odorous chemicals [1–3]. The symptoms are reactions to previous exposure that recur during subsequent exposure to the same or structurally unrelated chemicals at levels below those established to have harmful effects in the general population [2].

The prevalence of self-reported chemical intolerance (CI) in population-based surveys ranges from 8 to 33%, whereas that of physician-diagnosed MCS or reports of disabling consequences in the form of social and occupational disruptions is much lower, ranging from 0.5 to 6.3% [4]. The most pertinent contributing factor to the large variability in estimated prevalence is the wide variation in definitions for CI and MCS [4]. The Quick Environmental Exposure Sensitivity Inventory (QEESI) that Miller and Prihoda developed [5] is a validated screening scale for CI. Using the same cutoff scores for the QEESI provided a prevalence of CI of 8.2% in a Danish population sample [6] and 7.5% in a Japanese population sample [7].

The symptoms of MCS can be mild to disabling, and they affect several organs, especially the central nervous system, most frequently with patients complaining of headache, irritability, and cognitive dysfunctions; additionally, the musculoskeletal, respiratory, and digestive systems are also frequently involved [2,8–11]. No precise definition of MCS has been established and most definitions of MCS are qualitative, relying on subjective reports from patients and clinicians of distressing symptoms and environmental exposure [3,4].

Several studies in the past decade uncovered regional cerebral blood flow (rCBF) distribution abnormalities in patients with MCS using single-photon emission computed tomography (SPECT) [12] and positron emission tomography (PET) [13–15], especially while processing odorous substances. In particular, patients with MCS were demonstrated to peculiarly react to sensory stimuli, with activation of brain areas connected with motivational and emotional processing of the information. We also demonstrated activation in the prefrontal cortex (PFC) during olfactory stimulation [16] and in the orbitofrontal cortex (OFC) [17] following olfactory stimulation in patients with MCS using several different odorants using near-infrared spectroscopy (NIRS) imaging. These results suggest that prefrontal information processing associated with odor-processing neuronal circuits and memory and cognition processes from past hazardous chemical exposure play significant roles in the pathology of MCS [16,17]. These results also suggest that past strong exposure activates the PFC during olfactory stimulation in patients with MCS, and strong OFC activation persists after stimulus exposure [17]. Thus, the chemical-sensitive state of patients with MCS might remain due to repeated daily exposure, eventually leading to intolerance to odorous chemicals [17].

Recent studies regarding olfactory stimuli in patients with MCS evaluated their brain activities using PET more than 20 min after stimulus exposure for approximately 10 min [14,15]. However, the symptoms of patients with MCS often appear immediately after exposure to chemicals. Thus, evaluating brain activities during olfactory stimulation is important for clarifying the pathology of this disorder. NIRS is an optical technique that provides a noninvasive measure of continuous changes in rCBF during a task of the test. The spatial resolution of NIRS is inferior to that of other functional neuroimaging such as fMRI (functional magnetic resonance imaging), PET, and SPECT. However, NIRS has the advantage of a high time resolution and the feasibility of being performed under natural conditions. Thus, NIRS is suitable for monitoring rCBF during olfactory stimulation testing.

Past provocation studies identified no clear dose–response relationship between exposure and reaction in MCS [18]. Cullen defined MCS as an acquired disorder of recurrent symptoms, referable to multiple organ systems, occurring in response to chemically unrelated compounds at much lower doses than those established in the general population to cause harmful effects [1]. However, several provocation studies uncovered no objective differences between patients with MCS and healthy controls regarding their reactions to chemical exposures at air concentrations less than their health-based air quality guidelines and significantly less than their odor thresholds [19–22].

The aim of this study was to investigate the association of odor thresholds and changes in rCBF in the prefrontal area during olfactory stimulation at odor threshold levels in patients with MCS, using NIRS imaging. To the best of our knowledge, few studies have investigated this association using functional brain imaging in olfactory provocative testing.

## Methods

### Patients

Patients with MCS were diagnosed in the outpatient department (Outpatient Department of Sick House Syndrome, Hyakumanben Clinic, Kyoto, Japan) for people with chemical sensitivities between October 2009 and January 2014. The same definitions for MCS used in our previous studies [16,17] were applied in this study. MCS was diagnosed according to the 1999 consensus criteria [23]. As described in detail previously [16,17], patients diagnosed with chronic fatigue syndrome, fibromyalgia syndrome, or mental health disorders were excluded from the study. Patients with hyperpiesia, hyperlipidemia, diabetes, and/or allergic rhinitis were also excluded. The MCS condition of all patients was confirmed by the clinic physician during recruitment, which was conducted 3 months prior to olfactory stimulation testing using NIRS. Controls were recruited and selected to match the patients by age and sex at the group level. The same inclusion and exclusion criteria were applied for all patients and controls as those used in our previous studies [16,17]. Inclusion was based on QEESI scores, whereas the exclusion criteria included abnormal hematological examinations, smoking, drug or alcohol abuse, medications, pregnancy, and severe nasal stuffiness [16,17]. In addition, the subjects who confirmed olfactory disturbance in the olfactory ability test to be hereinafter described were excluded.

This study was approved by the ethical committee for human research at the Hyakumanben Clinic (99642–61). This study was also approved by the ethical committee for human research at the Louis Pasteur Centre for Medical Research (LPC.13). This study was performed according to the guidelines of the Declaration of Helsinki. All patients provided written informed consent and received the equivalent of 5000 JPY for their participation. This study was conducted from October 2013 to January 2014.

### Olfactory ability testing

Odor detection and recognition thresholds were determined using a T&T olfactometer (Daiichi. Yakuhin Sangyo, Ltd., Tokyo, Japan), which includes five odorants (odorant A [rose, light, and sweet], β-phenyl ethyl alcohol; odorant B [caramel, burning], methyl cyclopentenolone; odorant C [sweat], isovaleric acid; odorant D [fruits, heavy, and sweet], γ-undecalactone; and odorant E [vegetable chips, fecal], skatole). T&T olfactometry is the standard Japanese olfactory test, and it is routinely performed clinically [24,25]. Each odorant has eight degrees (log 10 serial dilutions) of concentrations (−2 to 5) excluding odorant B (−2 to 4). This test kit is used to determine of odor detection and recognition thresholds for each odorant [26,27].

The stimulus concentrations are presented in an ascending series and sniffed from strips of blotter paper dipped into the odorant solutions by the examiner before sampling by the subject. The concentration at which a stimulus was first noticed (but usually not recognized) was defined as the detection threshold, and the concentration at which the subject could identify the odor was defined as the recognition threshold. Thus, the detection threshold is defined as the lowest concentration detectable by the subject, whereas the recognition threshold is defined as the lowest concentration at which the odor can be identified. The means of the detection and recognition threshold values of the subcomponents of the test are used as the dependent measures [28]. The detection and recognition thresholds for five odorants were therefore averaged, and we used the values to evaluate olfactory acuities [27,29,30].

Odor recognition thresholds of 5.6 to 5.8, 1.1 to 5.5, and −2 to 1.0 were defined as ‘anosmia,’ ‘hyposmia,’ and ‘normosmia,’ respectively, as the Japanese Olfactory Test Committee agreed to use this criteria [31]. The detection and recognition thresholds approximately corresponded to 0 and 1, respectively, in Japanese people [29].

### Olfactory stimulation

In olfactory stimulation testing using NIRS imaging, the same T&T olfactometer was used in this study. To avoid a strong and cumulative body burden on patients with MCS, the olfactory stimulation test for each person was limited to total of six repetitions (two odorants and three concentrations). As odorants that were commonly perceived during ordinary daily activities, odorants D (γ-undecalactone [fruits, heavy, and sweet]) and E (skatole [vegetable chips, fecal]) were used in this study. The perception of these odors was assessed by placing the test strip of blotter paper dipped into the odorant solutions at a distance of approximately 10 mm from the noses of both patients with MCS and controls. The concentration levels of the odorants were set at the odor recognition threshold (1) or normally perceived odor level (4), with a non-odorant control (strip of blotter paper without odorant) used as placebo.

### Experimental procedure

**Fig 1:**
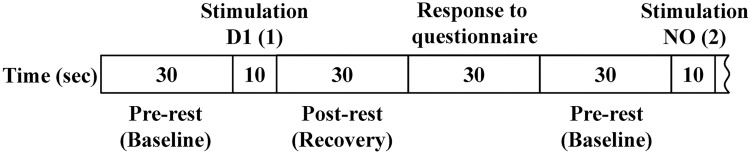
Experimental protocol. First, the subjects had a 30-s pre-rest. Then, the subjects were given an olfactory stimulus for 10 s, followed by a 30-s post-rest period and a 30-s period to complete a questionnaire on odor intensity and subjective assessments of hedonic and irritating odors. The 130-s cycles for each odorant were repeated six times consecutively.

In the present study, we followed the same experimental procedure (prior interviews, conditions of test room and subjects, and experimental protocol) with our previous studies. As described in detail previously [16,17], subjects sat in a comfortable chair and remained in the test room (temperature of approximately 22°C) for a sufficient period to feel comfortable before being exposed to the odorants. During the experiments, the subjects closed their eyes and slowly repeated the Japanese alphabet in an undertone to establish a stable rCBF prior to olfactory stimulation. They stopped repeating the Japanese alphabet and closed their eyes during olfactory stimulation, which lasted for 10 s. Olfactory stimulation was performed after a 30-s pre-rest period to establish the baseline level (Fig 1). The questionnaire (30 s) on odor intensity and hedonic and irritating scales was completed immediately after a 30-s rest period (post-rest) to allow recovery after olfactory stimulation (Fig 1). Afterward, the same process was repeated for an additional five olfactory stimuli. Odor intensity was rated on a six-point Likert scale ranging from not at all (0) to strong (5). Hedonic response was rated on a nine-point Likert scale ranging from discomfort (−4) to comfort (4). Irritation was evaluated on a visual analog scale, with responses ranging from “not at all” to “strong.”

Olfactory stimuli were applied in the following order: D1, non-odorant (NO), E1, D4, NO, and E4. The 130-s cycles were repeated six times consecutively. Thus, the order of the six repetitions (1–6) was as follows: D1 (1), NO (2), E1 (3), D4 (4), NO (5), and E4 (6).

### NIRS data acquisition

Changes in oxygenated hemoglobin (oxyHb) concentrations in the prefrontal area were measured using the LABNIRS Optical Multi-channel Monitor functional NIRS topography system (Shimadzu Corporation, Kyoto, Japan). Local blood flow to the relevant brain regions increases and oxygenated blood displaces deoxygenated blood when neurons become active. These changes reflect neuronal activity as their levels correlate with evoked changes in rCBF [32–34]. Pairs of illuminators and detectors were set 3 cm apart in a 3 × 11 lattice pattern to form 52 channels through a holder set in the prefrontal area. Changes in the oxyHb concentration were recorded every 54 ms using the NIRS system. Optical data were analyzed on the basis of the modified Beer–Lambert Law and signals reflecting the oxyHb concentration changes in an arbitrary unit were calculated (millimolar–millimeter) [35].

### Self-reported physical and psychological status

Participants completed a same self-reported questionnaire with our previous studies for the assessment of physical and psychological parameters. As described in detail previously [16,17], the questionnaire included the Chemical Sensitivity Scale for Sensory Hyper-reactivity (CSS-SHR), the Somato-Sensory Amplification Scale (SSAS), the Autonomic Perception Questionnaire (APQ), the Tellegen Absorption Scale (TAS), the Marlowe–Crowne Social Desirability Scale, the Taylor Manifest Anxiety Scale (TMAS), the Negative Affectivity Scale (NAS), and the Toronto Alexithymia Scale (TAS-20) that evaluates the total score and the scores of the three subscales, which assess difficulties in identifying feelings (DIF), difficulties in describing feelings (DDF), and externally-oriented thinking (EOT).

### Statistical analyses

OxyHb levels during olfactory stimulation were compared with those during the pre-rest period as a baseline level in each channel for evaluating the effects of olfactory stimulation on brain activity [16]. The oxyHb concentrations between the 30-s rest period after olfactory stimulation and the baseline during the pre-rest period were also compared in each channel [17]. The raw data from each channel were converted into z-scores [16,17]. Following the Shapiro–Wilk normality test and the covariate test for age, the *t*-test was used to compare brain activity obtained from NIRS imaging for all channels between patients with MCS and controls and was applied to analyze the results of self-reported physical and psychological scales to determine differences between the two groups at baseline. The nonparametric Mann–Whitney U-test was used to analyze the results of the odor thresholds and olfactory stimulation questionnaires and quantify the differences between two groups. All data analyses were performed using SPSS statistics software, version 23.

## Results

### Participants

The participants included 12 patients with MCS (age, 29–65 years; mean, 53.7 ± 9.9 years; all females) and seven controls (age, 27–55 years; mean, 45.3 ± 9.3 years; all females). Two patients with MCS did not fulfill the inclusion criterion for QEESI scores, one of whom had a severe runny and stuffy nose on an experimental day. One control exhibited a high immunoglobulin E level on hematological examinations. The remaining 10 nonsmoking patients with MCS (age, 48–65 years; mean, 56.4 ± 6.6 years; all females) and six nonsmoking controls (age, 27–55 years; mean, 44.7 ± 10.0 years; all females) met all the criteria, and they were included in the analyses. Demographic characteristics of the study population are depicted in Table 1. All patients with MCS attempted to avoid exposure to odorous chemicals as much as possible. These patients were homemakers or pensioners. Their occupations included three clerical employees in offices, a supermarket baker, a teacher, and a fabric tinter. Three controls also attempted to avoid exposure to odorous chemicals as much as possible. Their occupations were as follows: a teacher, a company executive, and a homemaker whose previous occupation was hospital dietician. Three controls did not consciously seek to avoid exposure to odorous chemicals, and their occupations were a medical coding staff, a teacher, and a graduate student specializing in clinical psychology.

**Table 1:** Demographic characteristics of the study population.

	MCS (*n* = 10)	Controls (*n* = 6)
Sex, male/female, (*n*)	0/10	0/6
Age, mean ± SD, (years)	56.4 ± 6.6	44.7 ± 10.0
Occupation, (*n*)		
Homemakers or pensioners	4	1
Clerical employees in offices	3	
Supermarket bakers	1	
Teachers	1	2
Fabric tinters	1	
Company executives		1
Medical coding staff		1
Graduate students		1

### Odor thresholds

**Fig 2:**
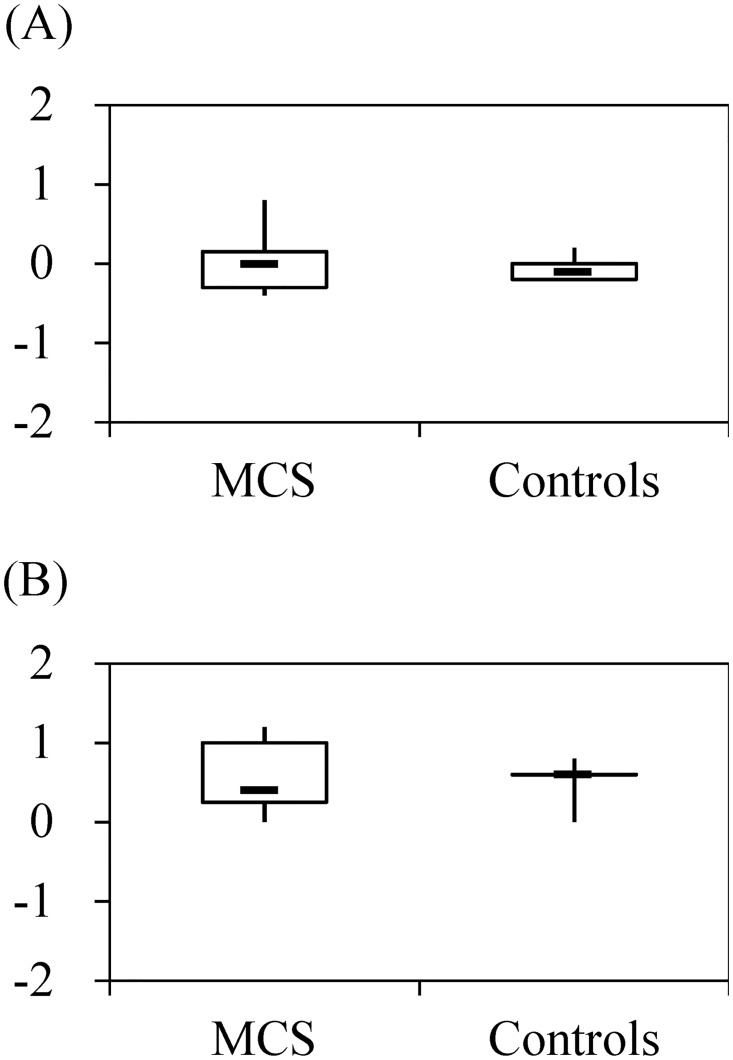
Detection thresholds (A) and recognition thresholds (B) by patients with multiple chemical sensitivity (MCS) (*n* = 10) and controls (*n* = 6) in olfactory ability testing. *Y*-axes present the mean values of five odorants.

The median T&T detection threshold score for patients with MCS was 0.0 (range, –0.4 to 0.8) and was –0.1 (range, –0.2 to 0.2) for controls; the difference was not statistically significant. The median T&T recognition threshold score for patients with MCS was 0.4 (range, 0.0 to 1.2), versus 0.6 (range, 0.0 to 0.8) for controls. This difference was also not statistically significant (Fig 2).

### NIRS imaging and subjective evaluation of odors

**Fig 3:**
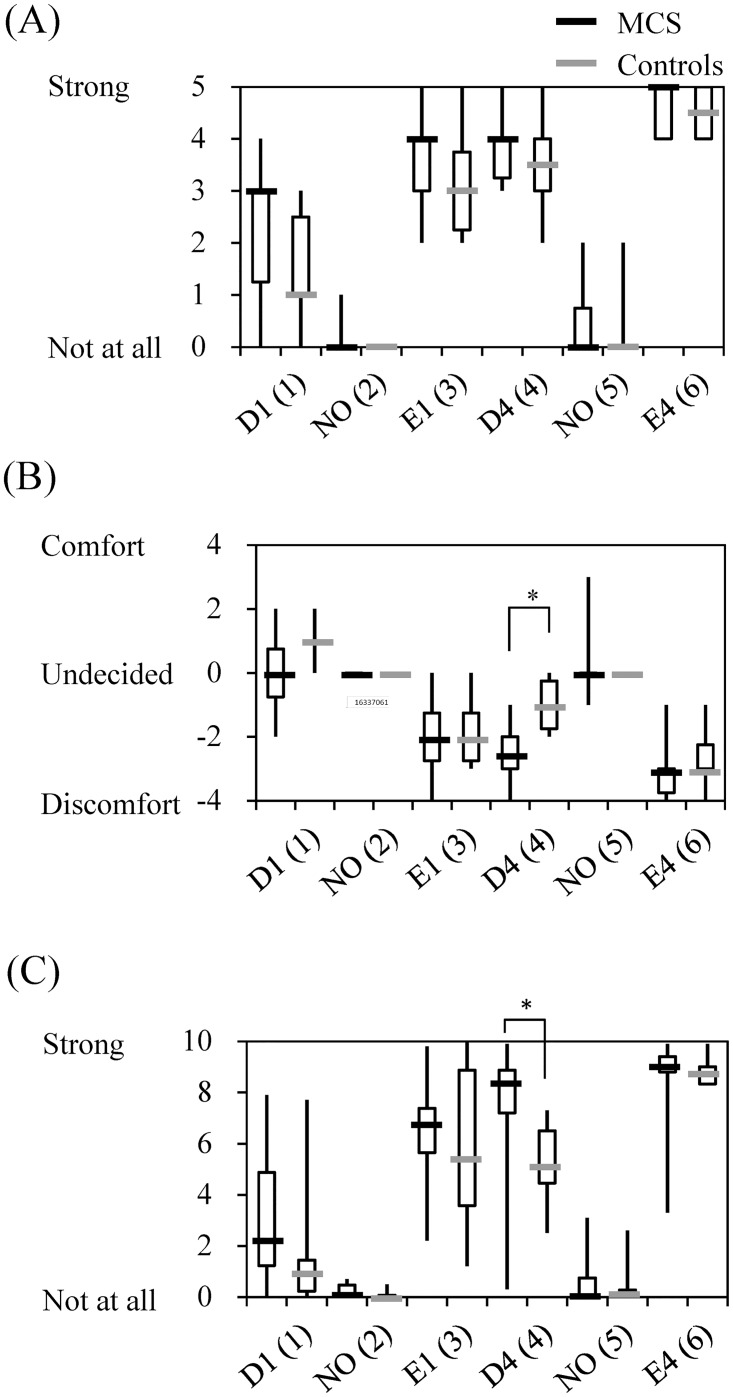
Rating of odor intensity (A) as well as hedonic (B) and irritating odors (C) by patients with multiple chemical sensitivity (MCS) (*n* = 10) and controls (*n* = 6) after olfactory stimulation. Abbreviations: D1, γ-undecalactone and concentration level 1; D4, γ-undecalactone and concentration level 4; E1, skatole and concentration level 1; E4, skatole and concentration level 4; and NO, non-odorant. Numbers in parentheses in column 1 indicate the order of the six repetitions (1–6). Statistically significant differences between groups are indicated: *p < 0.05.

Data for subjective odor intensity and assessments of hedonic and irritating odors by patients with MCS and controls after olfactory stimulation are shown in Fig 3. In the subjective evaluation, both patients with MCS and controls responded “not at all” on the odor intensity scale, “undecided” on the hedonic scale, and “not at all” on the irritation scale for both NO (2) and NO (5). These differences between patients with MCS and controls were not statistically significant.

The odor intensity scores of patients with MCS after exposure to odorants were higher (sense of stronger) than those of controls, but the differences were not significant. The hedonic scores of patients with MCS after exposure to odorants were lower (sense of more discomfort) than those of controls. The difference for D4 (4) was statistically significant. The irritation scores for patients with MCS after exposure to odorants were higher (sense of stronger) than those of controls. The difference for D4 (4) was also statistically significant. The differences for E4 (6) were not significant on either the hedonic or irritation scales, but saturation of the scores was assumed to be causally related to the results. Overall, odorant E was associated with stronger odor intensity, more discomfort, and greater irritation than was odorant D.

**Fig 4:**
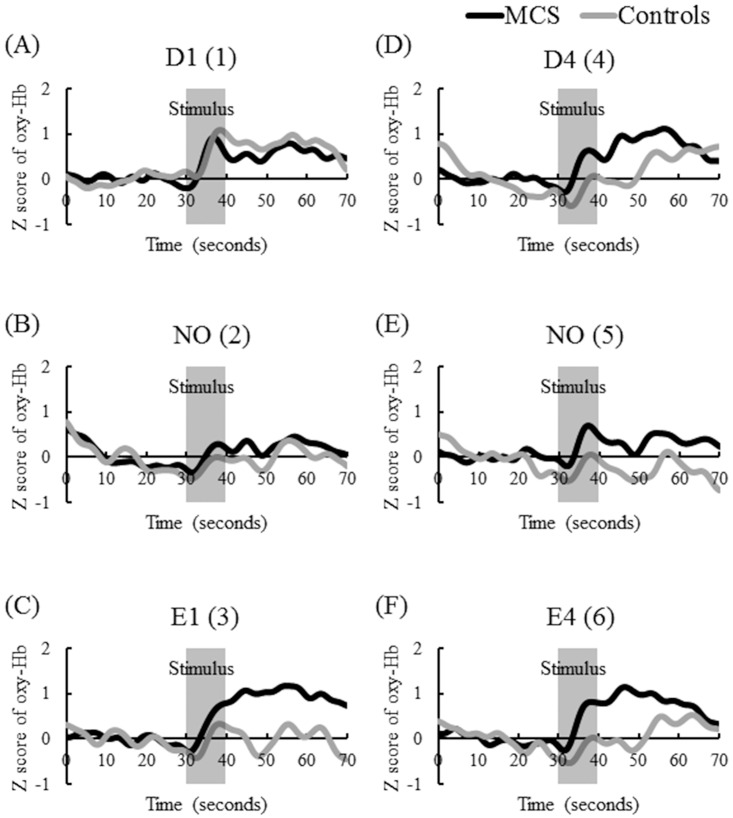
Time-course of average z-scores of all channels for oxygenated hemoglobin (oxyHb) levels in patients with multiple chemical sensitivity (MCS) (*n* = 10) and controls (*n* = 6) during pre-rest (baseline, 10–30 s), stimulation (30–40 s), and post-rest (recovery, 40–70 s). *Y*- and *X*-axes present the z-scored oxyHb values and times, respectively. Signals reflecting the oxyHb concentration changes in arbitrary units were calculated (millimolar–millimeter). The signal data were adjusted using a fast Fourier transform filter smoothing technique (OriginPro 2016 software of OriginLab Corporation). The cutoff frequency was determined at 35 points. The MCS group is indicated as a black line, and the control group is indicated as a gray line. Abbreviations: D1, γ-undecalactone, and concentration level 1; D4, γ-undecalactone, and concentration level 4; E1, skatole, and concentration level 1; E4, skatole, and concentration level 4; and NO, non-odorant. Numbers in parentheses in column 1 indicate the order of the six repetitions (1–6).

**Fig 5:**
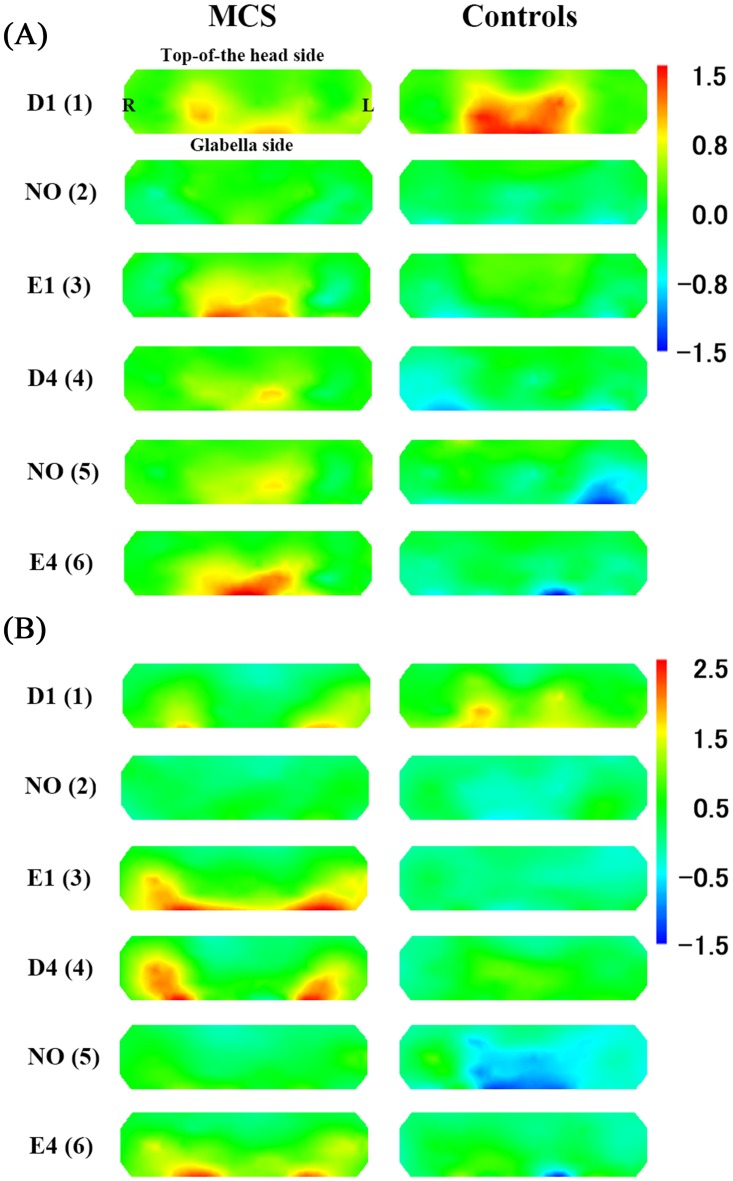
Topographical maps of average z-scores for oxygenated hemoglobin (oxyHb) levels between patients with multiple chemical sensitivity (MCS) (*n* = 10) and controls (*n* = 6). Abbreviations: D1, γ-undecalactone, and concentration level 1; D4, γ-undecalactone, and concentration level 4; E1, skatole, and concentration level 1; E4, skatole, and concentration level 4; and NO, non-odorant. Numbers in parentheses in column 1 indicate the order of the six repetitions (1–6).

The time-course of average z-scores of all channels for oxyHb in the MCS and control groups during pre-rest, stimulation, and post-rest are shown in Fig 4. The results of the *t*-test in terms of the average of all channels (1–52) comparing z-scores for oxyHb concentrations between patients with MCS and controls are shown in Table 2. Fig 5 provides the topographical maps of the average z-scores for oxyHb in patients with MCS and controls. Increases in rCBF levels in patients with MCS were suppressed after exposure to NO (2) and NO (5). There was no difference between patients with MCS and controls in responses to PFC. The trend of changes of rCBF for D1 (1) was not different between patients with MCS and controls. This response was observed in our previous studies, and it may have been caused by affective tension due to the first test [16,17]. For E1 (3), D4 (4), and E4 (6), larger increases in rCBF were observed in patients with MCS than those on controls, and the differences persisted approximately 20–30 s after olfactory stimulation. Significant differences in rCBF responses were observed between patients with MCS and controls during D4 (4) exposure and after E1 (3) exposure (Table 2). For E1 (3), D4 (4), and E4 (6), the activation (defined as greater increase in rCBF due to olfactory stimulation) areas of the prefrontal area during [16] and after olfactory stimulation [17] were similar to the results of our previous studies. These activations after olfactory stimulation in patients with MCS were especially strong in the lateral OFC (Fig 5).

**Table 2:** The *t*-test results for all channels (1–52) comparing z-scores for oxygenated hemoglobin levels between patients with multiple chemical sensitivity (MCS) and controls.

	Test	MCS (*n* = 10)	Controls (*n* = 6)	*p* value
Stimulus	D1 (1)	0.44 (0.48)	0.55 (0.59)	0.693
	NO (2)	−0.01 (0.31)	−0.20 (0.57)	0.468
	E1 (3)	0.27 (0.33)	−0.04 (0.62)	0.208
	D4 (4)	0.23 (0.32)	−0.28 (0.23)	0.004*
	NO (5)	0.27 (0.45)	−0.23 (0.51)	0.062
	E4 (6)	0.34 (0.36)	−0.27 (0.81)	0.054
Recovery	D1 (1)	0.56 (0.63)	0.77 (0.60)	0.524
	NO (2)	0.23 (0.62)	0.02 (0.50)	0.482
	E1 (3)	1.00 (0.75)	0.01 (0.56)	0.014*
	D4 (4)	0.79 (1.06)	0.34 (1.14)	0.431
	NO (5)	0.34 (0.62)	−0.30 (1.13)	0.166
	E4 (6)	0.82 (0.48)	0.18 (1.46)	0.221

Values are expressed as means (± standard deviations).

* Significant at *p* < 0.05.

Abbreviations: D1, γ-undecalactone and concentration level 1; D4, γ-undecalactone and concentration level 4; E1, skatole and concentration level 1; E4, skatole and concentration level 4; and NO, non-odorant. Numbers in parentheses in column 1 indicate the order of the six repetitions (1–6).

### Self-reported physical and psychological status

The results of the *t*-test for the physical and psychological scales are presented in S1 Table. QEESI and CSS-SHR scores were significantly higher for patients with MCS than for controls. APQ and TAS-20 DIF scores were significantly higher for patients with MCS than for controls. No significant differences were observed in the SSAS, TAS, MCSD, TMAS, NAS, TAS-20 total, TAS-20 DDF and TAS-20 EOT scores.

## Discussion

Double-blind provocation challenges in patients with MCS referred by physicians were performed in an environmental chamber and a variety of chemicals such as formaldehyde, natural gas, cleaners, and combusted kerosene were employed on the basis of the patient’s individual clinical histories. The exposure concentrations were not allowed to exceed existing threshold limit values, and clean air was used as a placebo or sham control. None of the patients demonstrated a reliable response pattern across a series of challenges [19]. Double-blind, placebo-controlled provocation studies in patients with subjective MCS and matched controls uncovered no objective differences between the groups regarding their reactions to chemical exposure. The subjects were exposed to a mixture of hydrocarbons at the air concentrations set below an experimentally determined odor threshold [20]. Similar results in single-blind provocation studies for patients with MCS have been reported using formaldehyde and toluene at air concentrations set below their indoor air quality guidelines and far below their odor thresholds [21,22].

In our study, single-blind provocation challenges were performed during olfactory stimulation at the odor threshold level and normal perceived odor level. Although the differences between patients with MCS and controls in both odor detection and recognition thresholds were not significant, greater increases in rCBF were observed in patients with MCS than in controls during olfactory stimulation at the recognition threshold or normally perceived level, and the differences persisted approximately 20–30 s after olfactory stimulation. The activation areas were the PFC during olfactory stimulation and the OFC after olfactory stimulation. Our results therefore suggest that the symptoms of MCS are not the reactions to chemical exposure at levels far below the odor thresholds, and the reflexive brain response to odor perceived instead involves the physical reactions of patients with MCS. When the odor recognition threshold exceeds the health-based threshold (i.e., no-observed adverse effect level), the air concentration of chemicals at the odor recognition threshold has harmful effects in the general population. However, when the health-based threshold exceeds the odor recognition threshold, the air concentration of chemicals at the odor recognition threshold is lower than those established to have harmful effects in the general population.

Olfactory input has direct connections via the olfactory bulb and primary olfactory (piriform) cortex to the amygdala and hippocampus. From these areas, sensory information is conveyed to the secondary olfactory cortices composed of the OFC and insular cortex [36]. Human episodic memory is the long-term memory process that enables one to mentally and consciously relive specific personal events from the past [37,38]. The PFC regulates the formation and control of memory [39]. In particular, the PFC has been linked to cognitive control processes such as selection, engagement, monitoring, and inhibition in long-term memory [40], and it plays an important role in long-term odor memory [41,42]. Odors are powerful cues that trigger episodic memories. Episodic odor memory has extremely little long-term loss compared with memories of pictures or odor presented in a laboratory environment [43]. Among all sensory stimuli, odors appear to trigger the most vivid and emotional memories [44]. For the sense of smell, emotional processing related to stimulation or discomfort prevails in comparison to verbal or writing processing, and the consequence of the processing rapidly appears [36,43].

Patients with MCS exhibit stronger physical reactions to odors at normally perceived levels in daily life than healthy people do. The status persists due to repeated daily exposure to the odors, and they exhibit physical intolerance to odorous chemicals at levels less than those established to have harmful effects in the general population. Our results including the present study suggested that the change of odor information processing associated with odor-processing neuronal circuits and memory and cognition processes due to prior hazardous chemical exposure play significant roles in the chemical-sensitive state of patients with MCS [16,17]. The present study indicated that activation of rCBF in the PFC and OFC in patients with MCS could be observed during exposure to odors at the recognition threshold level. In this study, the first symptoms were triggered by the initial exposure to chemicals in seven patients with MCS. These included the use of organic solvents, pesticides or incense in the workplace; the use of diesel machines in the neighborhood; or chemical exposure after renovation of a house or moving into a newly built house. Three patients had episodes of repeated exposure to solvents emitted from a neighboring industrial plant or paint store or fragrances or pesticides emitted in the neighborhood. Patients with MCS subsequently complained about a chemical sensitive condition.

The dorsal part of the anterior cingulate cortex (ACC) is connected to the PFC, and it plays an important role in processing top-down and bottom-up stimuli and assigning appropriate control to other areas in the brain [45]. Thus, the past exposure event was stored as memories in these cortices through olfactory nerve circuits, the processing of top-down stimuli from these cortices involves the central system related to emotional and autonomic nervous system, and various physical or psychological symptoms would be induced in patients with MCS. The psychological evaluations in the present study indicated that scores in MCS patients were significantly higher than those in controls on the APQ and TAS-20 DIF scales. These results also may support the theory of response regulation by memory in the PFC. Andersson et al furthermore suggested the involvement of a limbic hyperreactivity and speculatively described the sensitivity with MCS as an inability to inhibit salient external stimuli in MCS [46].

Near-infrared rays sent out from the NIRS device can provide visual access to the cerebral cortex within approximately 20 mm from the scalp, but cannot access the deep portion of a cerebral limbic system. The NIRS has the advantage of a high time resolution and the feasibility of being performed under natural conditions compared with other functional neuroimaging methodologies such as fMRI, PET, and SPECT, which can access the cerebral limbic system. However, the connections between the cerebral cortex and cerebral limbic system, their odor information processing, and their associations with MCS symptoms are important to clarify the pathology of this disorder. Further research is necessary regarding these connections and their associations with the symptoms using the NIRS and these other methodologies during olfactory stimulation.

Our study had some limitations. First, the small sample size makes the results vulnerable to selection bias. This could be alleviated by including a larger study population. However, differences between the patients with MCS and the controls regarding the NIRS imaging data were evident and supported by similar findings in the ACC [13], PFC [16], and OFC [17] in previous studies. Second, to the best of our knowledge, this is the first case-control study investigating the association of odor thresholds and changes in rCBF in prefrontal areas during olfactory stimulation at the odor threshold level in patients with MCS using NIRS imaging. Further evaluation using several odorants associated with a wide range of levels of comfort/discomfort or weak/strong irritation for MCS would provide valuable information for understanding the pathology of this disorder. A third limitation was the lack of standardized objective measures to identify and define MCS. Most definitions of MCS are entirely qualitative, relying on subjective reports of distressing symptoms and environmental exposure from patients and physicians. Therefore, several participants were excluded on the basis of QEESI scores, hematological data, and the discretion of the clinic physician due to conditions such as mental or chronic disorders.

In conclusion, despite the small sample size, this experimental case-control study demonstrated that significant differences between patients with MCS and controls regarding odor thresholds were not observed, and larger increases in rCBF in the PFC and OFC were observed in patients with MCS than in controls in response to the olfactory stimuli at the odor recognition threshold or normally perceived odor level. These brain responses may involve cognitive and memory processing systems during past exposure to hazardous chemicals. Further research regarding the mode of action of chemical sensitivity through the cerebral limbic system due to chemicals that were recognized as harmful or hazardous during the past exposure event is needed.

## Supporting Information

S1 Table Results of the *t*-test for the physical and psychological scales. (DOCX)
