# Supplementary material for: Association of Odor Thresholds and Responses in Cerebral Blood Flow of the Prefrontal Area during Olfactory Stimulation in Patients with Multiple Chemical Sensitivity
Source: PLoS One. 2016 Dec 9;11(12):e0168006. doi: 10.1371/journal.pone.0168006 (PMC5148047; doi:10.1371/journal.pone.0168006)
Supplement: S1 Table — (DOCX) [file pone.0168006.s001.docx]

Online supporting information for the following article published in *PLoS ONE*

**Association of odor thresholds and responses in cerebral blood flow of the prefrontal area during olfactory stimulation in patients with multiple chemical sensitivity**

Kenichi Azuma^*^, Iwao Uchiyama, Mari Tanigawa, Ikuko Bamba, Michiyo Azuma, Hirohisa Takano, Toshikazu Yoshikawa, Kou Sakabe

** Corresponding Author: Department of Environmental Medicine and Behavioral Science, Kindai University Faculty of Medicine, Osakasayama, Osaka, Japan*

**Table S1.** Results of the *t*-test for the physical and psychological scales.

| Scales | MCS (*n* = 10) | Controls (*n* = 6) | *p* value |
| --- | --- | --- | --- |
| QEESI (CI) | 72.8 (10.5) | 17.2 (27.0) | 0.003* |
| QEESI (OI) | 26.4 (15.2) | 2.8 (3.4) | <0.001* |
| QEESI (SS) | 60.1 (16.5) | 12.0 (12.3) | <0.001* |
| CSS-SHR | 49.6 (3.3) | 33.3 (4.0) | <0.001* |
| SSAS | 34.7 (5.9) | 35.5 (4.8) | 0.783 |
| APQ^a^ | 143.8 (49.4) | 75.3 (26.2) | 0.004* |
| TAS | 10.4 (4.5) | 8.8 (7.4) | 0.606 |
| MCSD^a^ | 14.4 (4.4) | 17.3 (6.7) | 0.333 |
| TMAS | 11.9 (4.3) | 7.7 (5.0) | 0.092 |
| NAS | 35.3 (9.2) | 28.5 (7.8) | 0.155 |
| TAS-20 total | 48.6 (13.4) | 44.8 (4.0) | 0.518 |
| TAS-20 DIF | 14.9 (5.9) | 9.2 (2.2) | 0.041* |
| TAS-20 DDF | 12.9 (3.4) | 13.0 (2.5) | 0.952 |
| TAS-20 EOT | 20.8 (5.2) | 22.7 (3.2) | 0.441 |

Values are expressed as means (± standard deviations).

^*^ Significant at *p* < 0.05.

^a^ Because of missing values, *t*-test results included the following numbers of participants. MCS and control: APQ and MCSD, *n* = 9 and *n* = 6.

Abbreviations: CI, chemical intolerance; OI, other intolerance; and SS, symptom severity.
